# Supplementary material for: Onchocerciasis-associated epilepsy in Maridi, South Sudan: Modelling and exploring the impact of control measures against river blindness
Source: PLoS Negl Trop Dis. 2023 May 26;17(5):e0011320. doi: 10.1371/journal.pntd.0011320 (PMC10249816; doi:10.1371/journal.pntd.0011320)
Supplement: S1 Text — (Section A: Annotated input file; Section B: Plots of estimated sum-squared-values in the parameter space and corresponding prevalence obtained from model; Section C: Sensitivity Analysis and plots; Section D: Multivariate sensitivity analysis of MDA and vector control. (DOCX) [file pntd.0011320.s001.docx]

Supplementary information

**Title of the Manuscript**

Onchocerciasis-associated epilepsy in Maridi, South Sudan: modelling and exploring the impact of control measures against river blindness

**Authors**

Samit Bhattacharyya^1,3*^, Natalie V.S. Vinkeles Melchers^2^, Joseph N. Siewe Fodjo^3^,

Amit Vutha^4^, Luc E. Coffeng^2^, Makoy Y Logora^5^, Robert Colebunders^3^, Wilma A. Stolk^2^

**Authors affiliation:**

1. Department of Mathematics, School of Natural Sciences, Shiv Nadar Institution of Eminence, Dadri, UP, India.
2. Department of Public Health, Erasmus MC, University Medical Center Rotterdam, Rotterdam, The Netherlands.
3. Global Health Institute, University of Antwerp, Antwerp, Belgium.
4. Department of Mathematics, Ohio State University, Columbus, Ohio, 43210-1174, USA
5. National Neglected Tropical Disease Programme, Ministry of Health South Sudan, Juba, South Sudan

*[samit.b@snu.edu](mailto:samit.b@snu.edu),

**S1. Model version and input assumptions**

Programme and source code availability: ONCHOSIM was originally developed as a standalone computer Programme but is now incorporated as a disease-specific variant within WORMSIM, a generalized framework for modelling transmission and control of helminth infections in humans. For the current analysis, we used **WORMSIM version v2.78**. The Programme and source code are available at GitLab:

<https://gitlab.com/erasmusmc-public-health/wormsim2.78src>.

The ONCHOSIM input file is an XML file that can be edited with any text editor or alternatively, with an XML editor (such as Oxygen XML Editor). A copy of an annotated input file is included below, showing the input assumptions as used in this study to simulate onchocerciasis transmission with WORMSIM version 2.78. The documentation is split into fragments that cover the different elements of the input file (gray-shaded boxes). Together, these fragments constitute a complete input file. The following elements are distinguished:

- *Input file header*
- *Simulation*
- *Demography*
- *Onchocerciasis associated epilepsy*
- *Exposure*
- *Immunity*
- *Worm*
- *Fly*
- *Mass.treatment*
- *Vector.control*

Meaning of the formatting of the input files: Text formatted in green as **<!—- this is a comment -->**denotes a comment. Grouping name tags for sets of input parameters are displayed in **blue**, while **red** indicates the specific parameters for which input is to be given. The actual inputs are found in the quotation marks, formatted in **purple**.

**1.1 *Input file header***

<?xml version="1.0" encoding="UTF-8"?>

<wormsim.inputfile xmlns:xsi="http://www.w3.org/2001/XMLSchema-instance"

xsi:noNamespaceSchemaLocation="wormsim.xsd" >

<!-- Input file for ONCHOSIM: -->

<!-- Wormsim v2.78 -->
<!-- Authors: Samit Bhattacharyya-->

***1.2 <Simulation>***

The <simulation> element specifies the start year of the simulation, the timing of surveys (i.e. output moments), the number of skin snips taken at each survey and the age classes for output.

<!-- General settings for simulation and simulation output -->

<simulation>

<!-- Number of skin snip taken per person -->

<surveillance nr.skin-snips="2" individual-output="false">

<!-- Timing of surveys -->

<!-- month 0 represents January 1st -->

<!-- see note regarding "delay" below -->

<start year="1995" month="0" delay="-2"/>

<stop year="2020" month="1"/>

<interval years="1" months="0" />

<!-- Upper bounds of age categories in output -->

<age.classes>

<age.class age.limit= "5"/>

<age.class age.limit="10"/>

<age.class age.limit="20"/>

<age.class age.limit="30"/>

<age.class age.limit="40"/>

<age.class age.limit="50"/>

<age.class age.limit="90"/>

</age.classes>

</surveillance>

<warming.up duration="200"/>

<!-- Upper bounds and weights of reference population for -->

<!-- age and sex-standardized output (OCP standard pop) -->

<standard.population>

<age.group age.limit= "5" n.males="1507" n.females="1475"/>

<age.group age.limit="10" n.males="1426" n.females="1419"/>

<age.group age.limit="20" n.males="2485" n.females="2387"/>

<age.group age.limit="30" n.males="1210" n.females="1661"/>

<age.group age.limit="40" n.males= "787" n.females="1073"/>

<age.group age.limit="50" n.males= "569" n.females= "558"/>

<age.group age.limit="90" n.males= "466" n.females= "635"/>

</standard.population>

</simulation>

***1.3 <Demography>***

The <demography> element defines life tables for the male and female population, the maximum population size (above which random persons will be removed), a fertility table, and the initial population size and age distribution. See comments below.

<!-- Demographic parameters of simulated population -->

<demography>

<!-- whenever the simulated population size exceeds -->

<!-- the specified maximum, a random fraction is removed -->

<!-- See note regarding "delay" below

<the.reaper max.population.size="17600" reap="0.05" delay="-3"/>

<!-- Survival represents cumulative survival probability -->

<!-- and is determined for unspecified ages by linear -->

<!-- interpolation of values for specified age limits -->

<life.table>

<survival age.limit= "5" male.survival="0.804" female.survival="0.804"/>

<survival age.limit="10" male.survival="0.772" female.survival="0.772"/>

<survival age.limit="15" male.survival="0.760" female.survival="0.760"/>

<survival age.limit="20" male.survival="0.740" female.survival="0.740"/>

<survival age.limit="30" male.survival="0.686" female.survival="0.686"/>

<survival age.limit="40" male.survival="0.594" female.survival="0.594"/>

<survival age.limit="50" male.survival="0.509" female.survival="0.509"/>

<survival age.limit="90" male.survival="0.000" female.survival="0.000"/>

</life.table>

<!-- Fertility rates represent probabilities for women to give birth -->

<!-- to one child in some year, given a woman's age. -->

<!-- Rates are assumed constant within each age category -->

<!-- and ages limits represent upper bounds of categories. -->

<!-- See note regarding "delay" below -->

<fertility.table delay="-4">

<fertility age.limit= "5" birth.rate="0"/>

<fertility age.limit="10" birth.rate="0"/>

<fertility age.limit="15" birth.rate="0"/>

<fertility age.limit="20" birth.rate="0.109"/>

<fertility age.limit="30" birth.rate="0.300"/>

<fertility age.limit="40" birth.rate="0.235"/>

<fertility age.limit="50" birth.rate="0.119"/>

<fertility age.limit="90" birth.rate="0"/>

</fertility.table>

<!-- Population size to start simulation with -->

<initial.population>

<age.group age.limit= "5" n.males="4" n.females="4"/>

<age.group age.limit="10" n.males="5" n.females="5"/>

<age.group age.limit="20" n.males="3" n.females="3"/>

<age.group age.limit="30" n.males="4" n.females="4"/>

<age.group age.limit="40" n.males="5" n.females="5"/>

<age.group age.limit="50" n.males="4" n.females="4"/>

<age.group age.limit="90" n.males="4" n.females="4"/>

</initial.population>

</demography>

***1.4 Onchocerciasis associated epilepsy***

The <disease.process> element defines the parameters for development of onchocerciasis associated epilepsy. This module is a redefined version of a previously developed morbidity module for skin disease and blindness in the ONCHOSIM model [ref 39 in main text]. In the current version, we specify a threshold of cumulative exposure to microfilaria, a maximum age of individual to develop epilepsy, and the effect of epilepsy on the remaining life expectancy.

<!-- Parameters for development of OAE. -->

<!-- When a person's cumulative exposure to mf exceeds a -->

<!-- threshold, a person is considered epileptic. -->

<!-- Individual variation in susceptibility is modeled by -->

<!-- letting the threshold vary between individuals, assuming a -->

<!-- Weibull distribution with some mean and shape "p1", -->

<!-- truncated at the specified bounds "min" and "max". -->

<disease.processes>

<disease.process name="Epilepsy" cause="mf" susceptibility.shape.param="0.1367"

max.age.for.accumulation="18">

<regression.rate fun.nr="1" a="0" b="0" c="-1"/>

<disease.stage name="stage-zero" treshold="0"/>

<disease.stage name="stage-one" treshold="721.1"/>

<symptom name="OAE"/>

</disease.stage>

</disease.process>

</disease.processes>

<symptom.defs>

<symptom.def name="OAE">

<pct-life-expectancy-reduction dist.nr="1" min="72.6" max="82.7" mean="77.65"/>

</symptom.def>

***1.5 <Exposure>***

The <exposure> element defines the parameters for the exposure of humans to a vector (or infectious reservoir) and the contribution of humans to the vector cloud (or infectious reservoir). See comments below.

<!-- Parameters for exposure to fly bites. -->

<exposure>

<!-- Initial force of infection to introduce infection -->

<!-- into the simulated population; duration in years. -->

<initial.foi duration="7.5" foi="4"/>

<!-- Parameters for individual exposure to fly bites, -->

<!-- depending on gender, age, and personal factors. -->

<male>

<!-- Age-dependent exposure, relative to mean exp -->

<!-- of adult males, assuming a linear increase -->

<!-- between age 0 and 20, after which relative exposure is 1.0 -->

<exposure.function fun.nr="1" a="0.05" c="1"/>

<!-- Individual variation in exposure related to -->

<!-- e.g. occupation and attractiveness to flies, -->

<!-- assuming a gamma distribution with mean one -->

<!-- and variation 1/p1 (shape and rate p1), -->

<!-- truncated by "min" and "max". -->

<exposure.index dist.nr="4" min="0" max="20" p1="4.283"/>

</male>

<female>

<!-- age-dependent exposure, relative to mean exp -->

<!-- of adult males, assuming a linear increase -->

<!-- between age 0 and 20, after which relative exposure is 1.0. -->

<!-- Exposure of females to fly bites is assumed -->

<!-- to be 70% of that of males. -->

<exposure.function fun.nr="1" a="0.035" c="0.7"/>

<!-- Individual variation in exposure related to -->

<!-- e.g. occupation and attractiveness to flies, -->

<!-- assuming a gamma distribution with mean one -->

<!-- and variation 1/p1 (shape and rate p1), -->

<!-- truncated by "min" and "max". -->

<exposure.index dist.nr="4" min="0" max="20" p1="4.283"/>

</female>

</exposure>

***1.6 <Immunity>***

The <immunity> element defines the (optional) development of host immunity. Immunity is not considered in the current ONCHOSIM model.

<!-- Parameters related to development of host immunity. -->

<!-- These are currently set such that no immunity develops. -->

<immunity>

<male alpha="0" beta="1">

<immunity.function fun.nr="0" a="1"/>

<immunity.index dist.nr="0" min="0" max="20"/>

</male>

<female alpha="0" beta="1">

<immunity.function fun.nr="0" a="1"/>

<immunity.index dist.nr="0" min="0" max="20"/>

</female>

</immunity>

***1.7 <Worm>***

The <worm> element defines parameters for worm lifespan, prepatent period, mating between M and F worms, age-dependent production of microfilaria, mf density per worm and skin dispersal.

<!-- Parameters for worm survival and mf production. -->

<!-- Mf lifespan in months, see note regarding "delay" below -->

<worm mf-lifespan="9" monthly.event.delay="+1">

<!-- Worm lifespan in years, allowing for variation -->

<!-- between worms, assuming a Weibull distribution with -->

<!-- mean 10 and shape 3.76, bounded by "min" and "max" -->

<lifespan dist.nr="3" min="0" max="50" mean="10" p1="3.76"/>

<!-- Pre-patent during which worms do not produce mf and -->

<!-- are not affected by ivermectin -->

<prepatent.period dist.nr="0" mean="1"/>

<!-- Number of months a female can produce mf with one -->

<!-- insemination, and number of females one male worm -->

<!-- can inseminate per month. -->

<!-- If there are more female worms than the total male -->

<!-- potential, every female has a probability of being -->

<!-- inseminated equal to N_mw/N_fm*male.potential. -->

<mating cycle="3" male.potential="100"/>

<!-- Mf production by female worms as function of worm -->

<!-- age minus pre-patent period; mf production at un- -->

<!-- specified ages is determined by linear interpolation -->

<age.dependent.mf-production>

<mf-production age.limit="0" production="1"/>

<mf-production age.limit="5" production="1>

<mf-production age.limit="20" production="0"/>

</age.dependent.mf-production>

<!-- Expected N_mf per worm in skin snip as product of -->

<!-- number of mf contributed per fully fecund worm and -->

<!-- random dispersal factor representing the distance -->

<!-- between a worm and site of skin snip, assuming an -->

<!-- exponential distribution, truncated by "min" and -->

<!-- "max". -->

<skin.mf-density.per.worm fun.nr="1" a="7.6" c="-1"/>

<skin.dispersal dist.nr="2" min="0" max="5"/>

<skin-snip.variability dist.nr="5"/>

<!-- Poisson distribution for observed number of mf in -->

<!-- one skin snip. -->

</worm>

***1.8 <Fly>***

The <fly> element defines parameters that determine the successful uptake and development of L1 larvae into infective L3 larvae and also determines the fly biting rate.

<!-- Probability that an mf taken up by a fly bite develops -->

<!-- into an L3 and is transmitted to another human, taking -->

<!-- account of the fly's gonotropic cycle, survival, and -->

<!-- duration and probability of an ingested mf developing -->

<!-- into an infective L3 and surviving up to the point of -->

<!—transmission. -->

<fly transmission.probability="0.07345">

<!-- Functional relation between uptake of mf and mf -->

<!-- density in the skin, assuming exponential saturation -->

<!-- to maximum level a with initial slope b and shape c -->

<L1-uptake fun.nr="3" a="1.2" b="0.0213" c="0.0861"/>

<!-- Seasonal pattern in monthly biting rates (mbr), -->

<!-- as observed in Asubende, Ghana. -->

<!-- In the simulation, actual biting rates for an -->

<!-- individual are calculated as product of monthly -->

<!-- biting rate in Asubende, a factor representing -->

<!-- the mean exposure in adult males in the simulated -->

<!-- village relative to Asubende ("relative biting -->

<!-- rate"), and all other factors related to gender, -->

<!-- age, and individual variation in exposure. -->

<!-- To produce some desired endemicity level in the -->

<!-- simulation, adjust the relative biting rate such -->

<!-- that mf prevalence or density (distribution) in -->

<!-- the population (output at the desired time point) -->

<!-- equals the desired value. -->

<!-- N.B. individual variation in exposure to fly bites -->

<!-- also determines mean and distribution of simulated -->

<!-- infection levels -->

<monthly.biting.rates relative.biting.rate="0.910">

<mbr month="1" rate="2670"/>

<mbr month="2" rate="2350"/>

<mbr month="3" rate="1500"/>

<mbr month="4" rate="1920"/>

<mbr month="5" rate="1940"/>

<mbr month="6" rate="1690"/>

<mbr month="7" rate="2630"/>

<mbr month="8" rate="3410"/>

<mbr month="9" rate="3010"/>

<mbr month="10" rate="3290"/>

<mbr month="11" rate="3750"/>

<mbr month="12" rate="2690"/>

</monthly.biting.rates>

</fly>

<!-- parameters for mass treatment -->

***1.9 <Mass.treatment>***

The <mass.treatment> element defines parameters for the timing of mass treatment rounds, individual compliance (permanent, temporary and age dependent), and effects of ivermectin on mature worms, mf production by F worms and on mf.

<!-- Parameters for mass treatment -->
<!-- The statement v58=“true” is included for technical reasons -->

<!-- It indicates that the mechanisms employed here is -->

<!-- the same as in the previously published WORMSIM variant 2.58Ap9-->

<mass.treatment v58="true">

<compliance.options>

<!-- Random fraction of population permanently not eligible for treatment due -->

<!-- to chronic illness and random fraction of population in which ivermectin -->

<!-- does not work due to diarrhoe (temporary effect) -->

<compliance name="default" fraction.excluded="0.00" fraction.malabsorption="0"

compliance.model="0">

<!-- Weights for age and sex-specific compliance, given some expected overall -->

<!-- coverage in the eligible population; weights are constant within age groups -->

<age.and.sex.specific.compliance age.limit= "5" male.compliance="0" female.compliance="0"/>

<age.and.sex.specific.compliance age.limit="10" male.compliance="1" female.compliance="1"/>

<age.and.sex.specific.compliance age.limit="20" male.compliance="1" female.compliance="1"/>

<age.and.sex.specific.compliance age.limit="30" male.compliance="1" female.compliance="1"/>

<age.and.sex.specific.compliance age.limit="40" male.compliance="1" female.compliance="1"/>

<age.and.sex.specific.compliance age.limit="50" male.compliance="1" female.compliance="1"/>

<age.and.sex.specific.compliance age.limit="90" male.compliance="1" female.compliance="1"/>

</compliance>

</compliance.options>

<!-- Timing of individual mass treatment rounds (one line per mass treatment round), -->
<!-- specifying year, month (0 represents January 1st), and population -->
<!-- coverage (fraction of total village pop including those not eligible for treatment)-->

<!-- Varying between simulated scenarios, see note regarding "delay" below -->

<treatment.rounds>

<treatment.round year="2000" month="0" drug="ivermectin" coverage="0.6" delay="-1"/>

<treatment.round year="2001" month="0" drug="ivermectin" coverage="0.6" delay="-1"/>

<treatment.round year="2002" month="0" drug="ivermectin" coverage="0.6" delay="-1"/>

<treatment.round year="2003" month="0" drug="ivermectin" coverage="0.6" delay="-1"/>

<treatment.round year="2004" month="0" drug="ivermectin" coverage="0.6" delay="-1"/>

<treatment.round year="2005" month="0" drug="ivermectin" coverage="0.6" delay="-1"/>

<treatment.round year="2006" month="0" drug="ivermectin" coverage="0.6" delay="-1"/>

<treatment.round year="2007" month="0" drug="ivermectin" coverage="0.6" delay="-1"/>

<treatment.round year="2008" month="0" drug="ivermectin" coverage="0.6" delay="-1"/>

<treatment.round year="2009" month="0" drug="ivermectin" coverage="0.6" delay="-1"/>

<treatment.round year="2010" month="0" drug="ivermectin" coverage="0.6" delay="-1"/>

<treatment.round year="2011" month="0" drug="ivermectin" coverage="0.6" delay="-1"/>

<treatment.round year="2012" month="0" drug="ivermectin" coverage="0.6" delay="-1"/>

<treatment.round year="2013" month="0" drug="ivermectin" coverage="0.6" delay="-1"/>

<treatment.round year="2014" month="0" drug="ivermectin" coverage="0.6" delay="-1"/>

<treatment.round year="2015" month="0" drug="ivermectin" coverage="0.6" delay="-1"/>

<treatment.round year="2016" month="0" drug="ivermectin" coverage="0.6" delay="-1"/>

<treatment.round year="2017" month="0" drug="ivermectin" coverage="0.6" delay="-1"/>

<treatment.round year="2018" month="0" drug="ivermectin" coverage="0.6" delay="-1"/>

<treatment.round year="2019" month="0" drug="ivermectin" coverage="0.6" delay="-1"/>

<treatment.round year="2020" month="0" drug="ivermectin" coverage="0.6" delay="-1"/>

</treatment.rounds>

<v58.drugs>

<!-- ivermectin efficacy, specified according to mechanisms version 2.58, -->
<!-- as permanent reduction in worm capacity to produce mf (cumulative effects -->

<!-- allowed), pattern of how mf production recovers over time (to a new, reduced

<!-- maximum level) and fraction of mf surviving each treatment -->

<v58.drug name="ivermectin" compliance="default"

include.prepatent.worms="true">

<v58.treatment.effects permanent.reduction.mf-production="0.349"

period.of.recovery="0.875"

shape.parameter.recovery.function="1.483"

fraction.killed="0">

<fraction.mf.surviving dist.nr="0" mean="0.0"/>

<!-- Variability in treatment effects (relative to mean, expected effect), -->

<!-- assuming a Weibull distribution with mean one and shape "p1" -->

<treatment.effect.variability dist.nr="3" mean="1.0" p1="2"/>

</v58.treatment.effects>

</v58.drug>

</v58.drugs>

</mass.treatment>

***1.10 <Vector.control>***

The <vector.control> element defines parameters for setting the effectivity of vector control during periods of vector control.

<!-- Parameters for vector control. -->

<!-- Effectivity is specified as relative reduction in biting rates. -->

<!-- If no vector control is desired in the simulation, set timing -->

<!-- outside the scope of the simulation (after the last survey). -->

<!-- Vector control is usually assumed to be highly effective -->

<vector.control>

<period start.year="2000" stop.year="2020" effectivity="0.6"/>

</vector.control>

<!-- In ONCHOSIM, some events may be scheduled at the same time. The attribute -->
<!-- "delay" specifies at what time an event takes place, relative to other events -->

<!-- planned at the same time. The order of events is currently: human births, the -->

<!-- reaper, survey, mass treatment, worm and mf generation and death. -->



**S2. Plots of estimated sum-squared-values in the parameter space and corresponding prevalence obtained from model.**

**
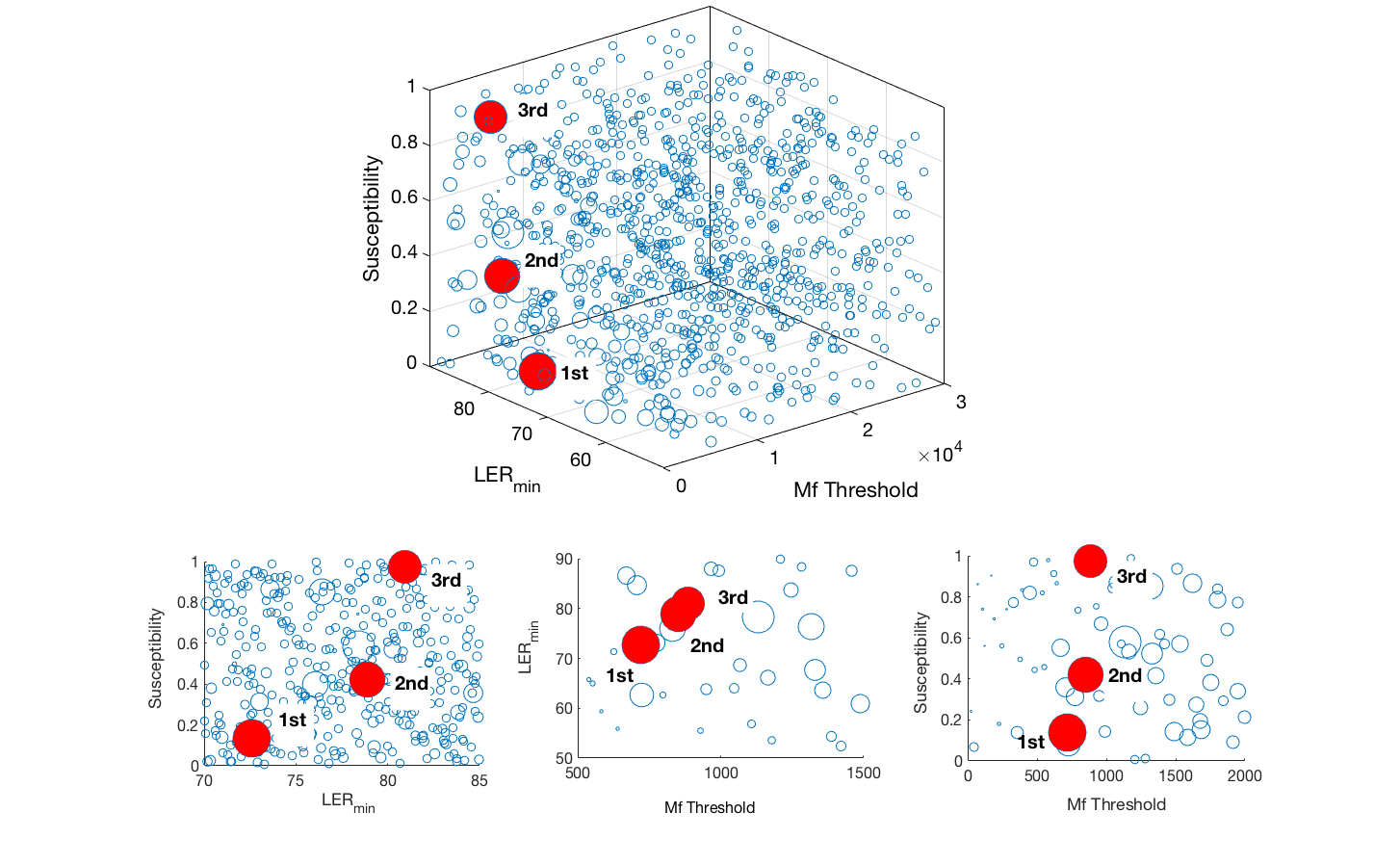
**

**Figure S1.** Figure represents the SSE (sum of squared errors) value in parameter estimation process depending on three parameters (a) susceptibility, (b) Mf threshold, (c) Life expectancy reduction (lower bound of the interval). ‘1st’, ‘2nd’ and ‘3rd’ in the figures denote the first three best estimates of sum squared errors in the parameters range.

**
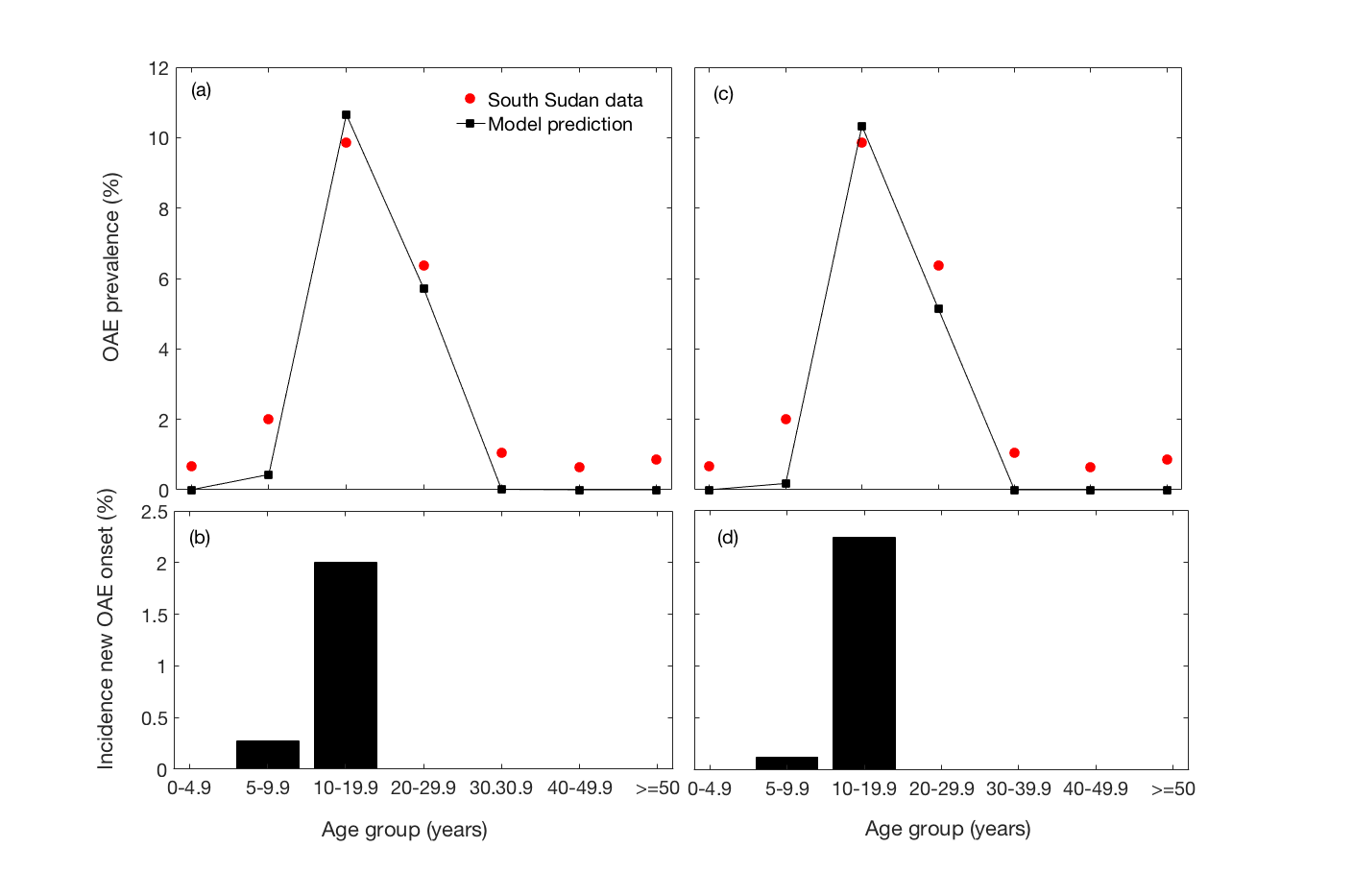
**

**Figure S2.** Figure represents the last two best fits of the model to the data (a - b) second best fit (*T* = 852.5, *S_i_* = 0.42, Mean *LER* = 83%), and (c-d) third best fit (*T* = 884, *S_i_* = 0.98, Mean *LER* = 85%). The first best fit is given in the main text (Fig. 2). For reference, see figure S1 in supplementary.


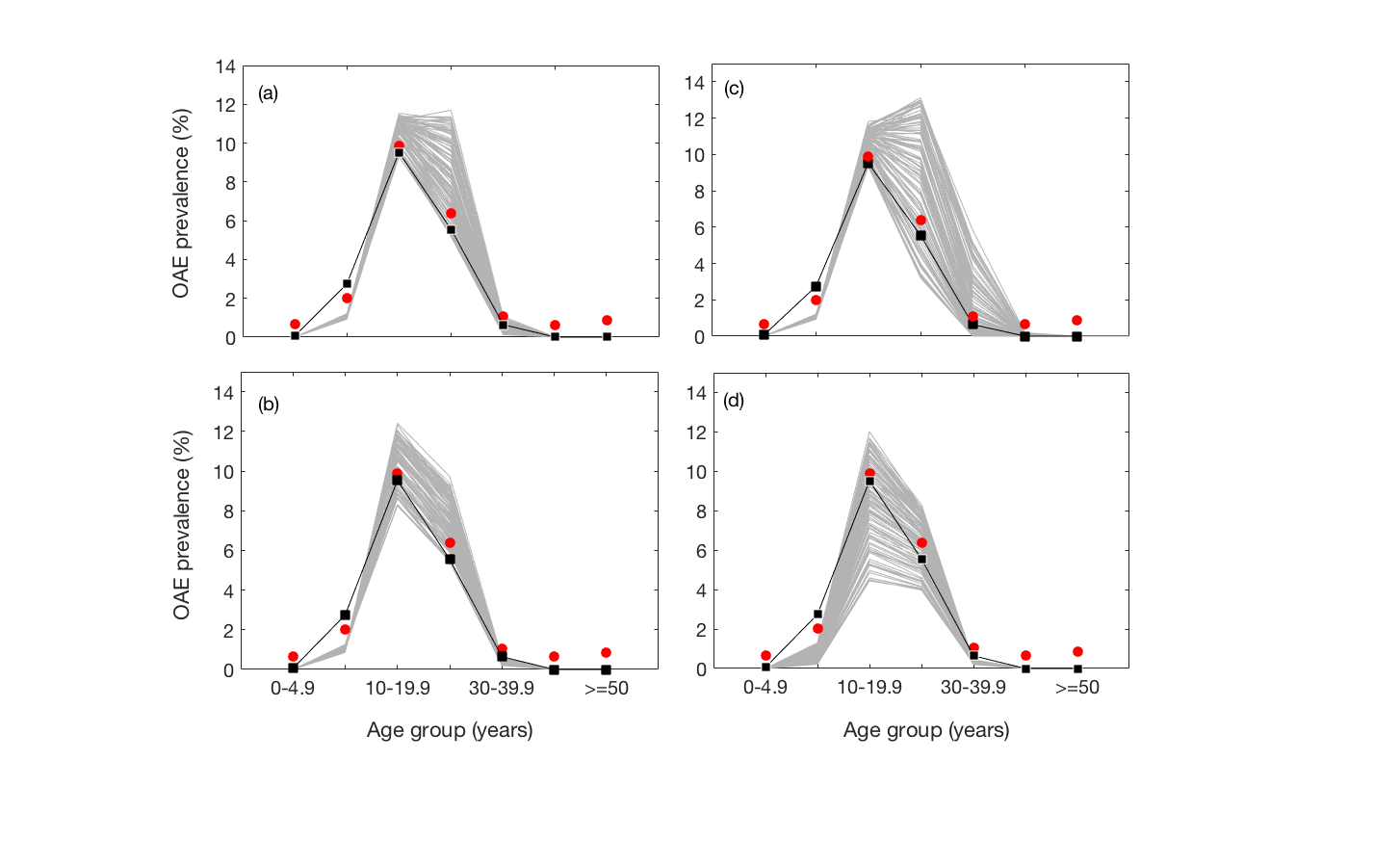
**S3.** **Sensitivity Analysis and plots**

**Figure S3:** Sensitivity of model predicted pattern of OAE-prevalence by age to univariate changes in model parameters: (a) parameter *a_max_*, (b) susceptibility parameter, (c) life expectancy reduction (LER) due to development OAE, (d) ABR. The red dots represent the observed age prevalence from Maridi, whereas the black line represents the best model fit. Light grey trajectories are model output for different values of respective sensitivity parameters. Each grey line is the mean of 100 runs.

**S4. Multivariate sensitivity analysis of MDA and vector control**

We performed multivariate sensitivity analysis to examine how sensitive simulated trends in total number of OAE cases are for changes in assumptions in the disease model parameter values. We used same Latin Hypercube sampling [LHS] technique in 3D-parameter space with following range: (i) inter-individual variation in individuals’ susceptibility for developing OAE - [0.075-0.2], (ii) reduction in life expectancy of epileptic individuals - [60%-80%], and (iii) the mf threshold - [600 - 800]. We draw 100 samples from the above range of the respective parameters, and run the model under different MDA coverage, and vector control. The results are presented in Fig S4, and S5.

**
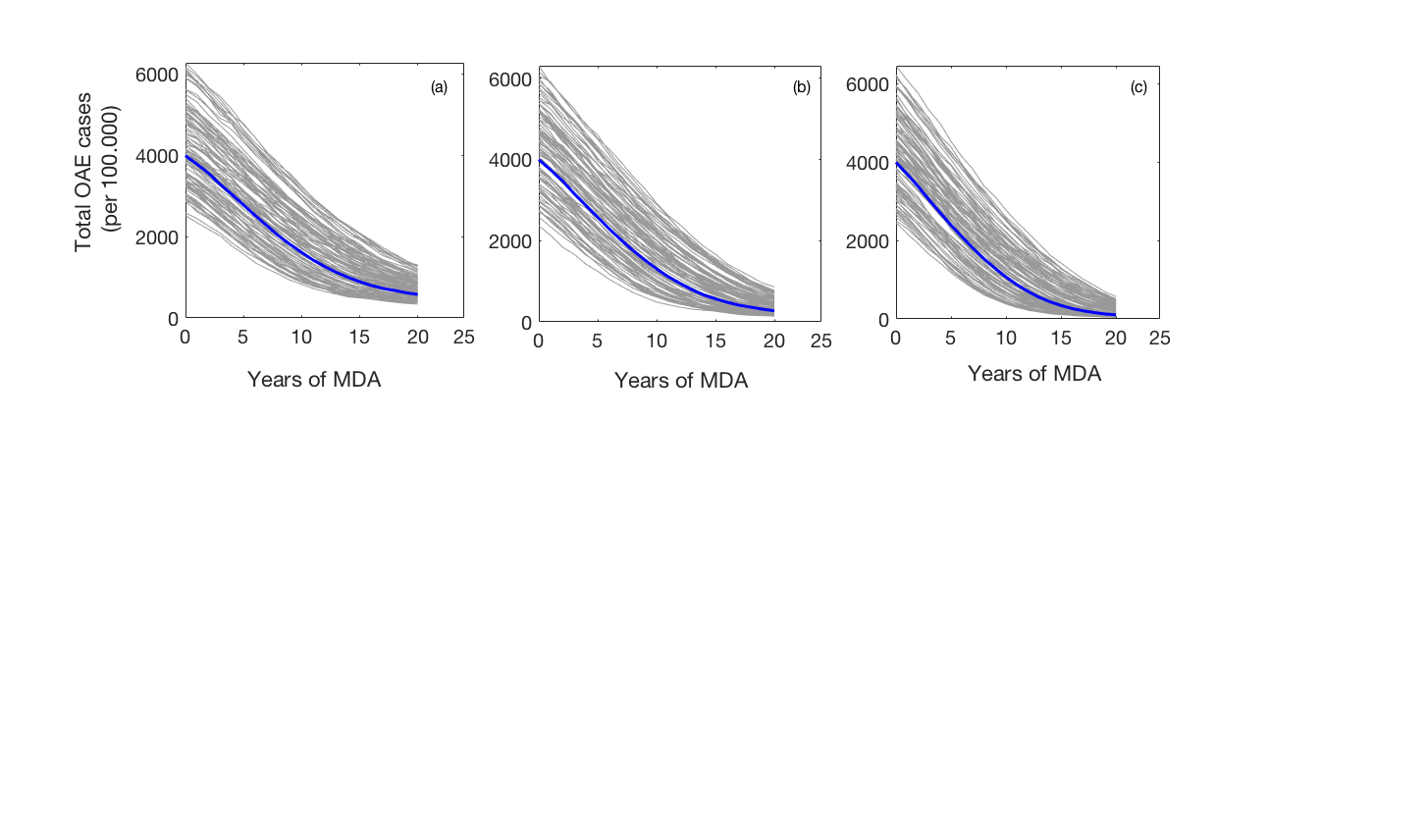
**

**Figure S4.** Sensitivity of model predicted decline in total number of OAE cases to multivariate changes in values of OAE model parameters during annual ivermectin MDA with coverage (a) 50%, (B) 60% and (c) 70%

**
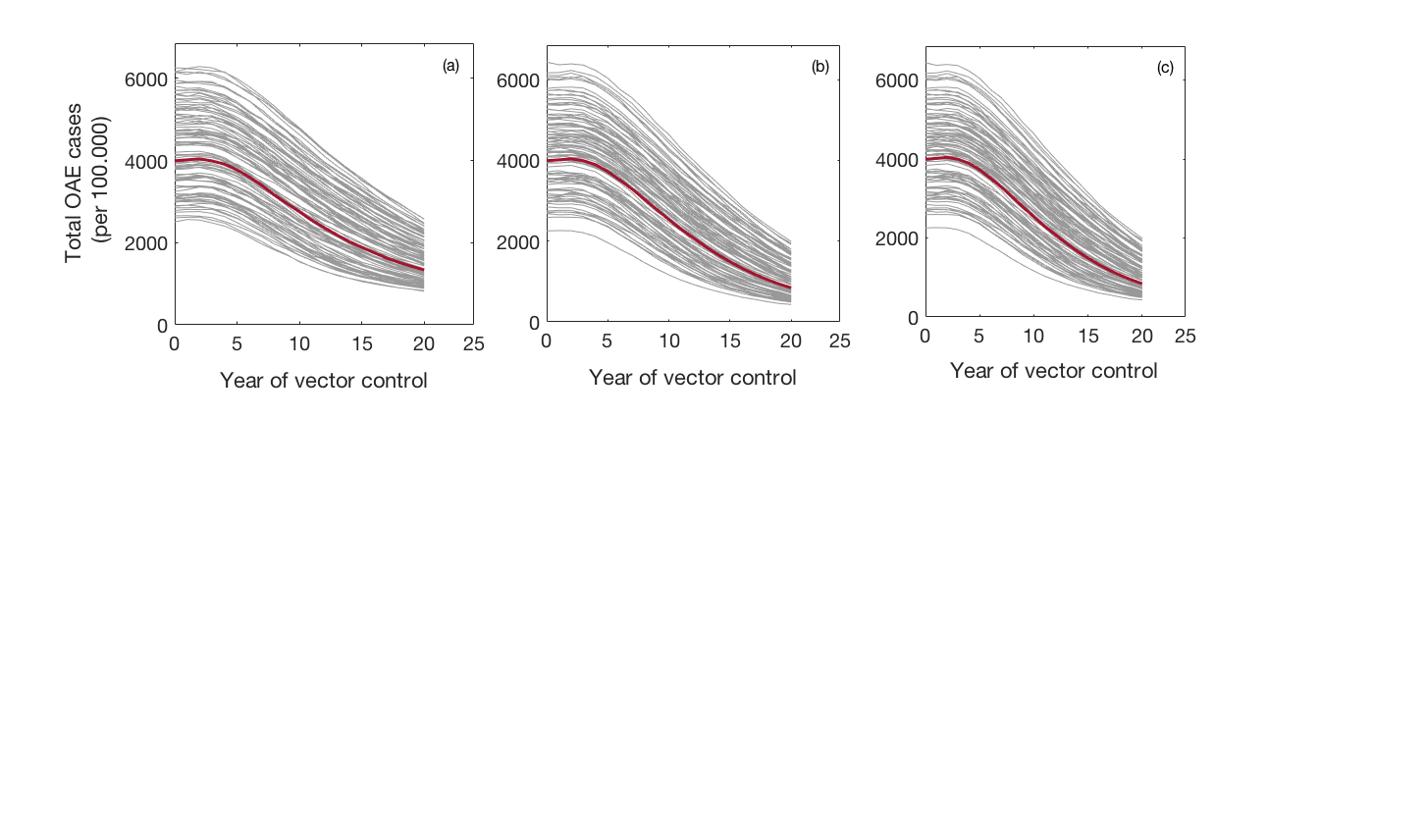
**

**Figure S5.** Sensitivity analysis of vector control with coverage (a) 60%, (B) 70% and (c) 80%


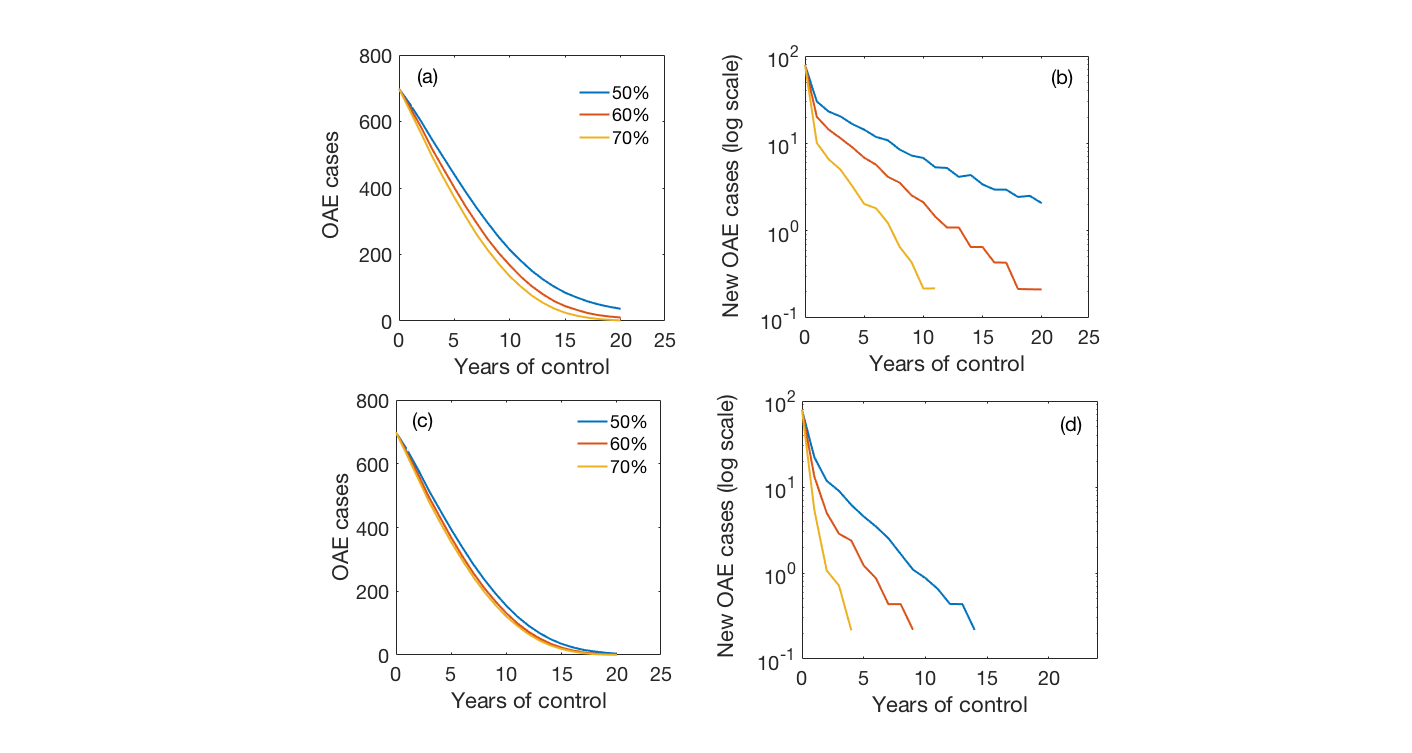


**Figure S6.** Predicted OAE cases under annual (a-b) and bi-annual (c-d) MDA treatment with different coverage. The ONCHOSIM model is calibrated with South Sudan data, and hence the total population size is around 18000.


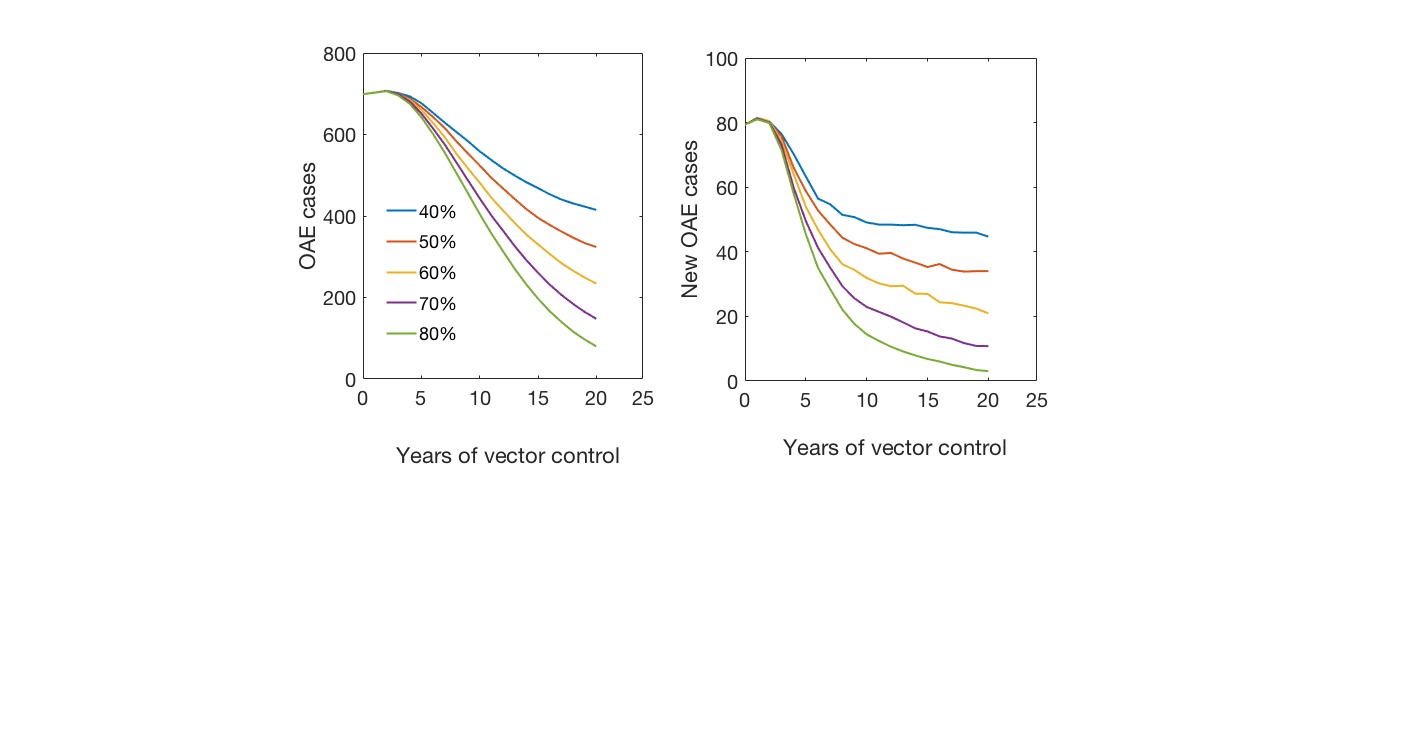


**Figure S7.** Predicted OAE cases and new cases under vector control with different coverage. The ONCHOSIM model is calibrated with South Sudan data, and hence the total population size is around 18000.
