# Supplementary material for: Onchocerciasis-associated epilepsy in Maridi, South Sudan: Modelling and exploring the impact of control measures against river blindness
Source: PLoS Negl Trop Dis. 2023 May 26;17(5):e0011320. doi: 10.1371/journal.pntd.0011320 (PMC10249816; doi:10.1371/journal.pntd.0011320)
Supplement: S1 Table — (DOCX) [file pntd.0011320.s002.docx]

S1 PRIME-NTD Table. Policy-Relevant Items for Reporting Models in Epidemiology of Neglected Tropical Diseases

Table: The Policy-Relevant Items for Reporting Models in Epidemiology of Neglected Tropical Diseases (PRIME-NTD) ^a^

| Principle ^b^ | What has been done to satisfy the principle? | Where in the manuscript is this described? |
| --- | --- | --- |
| 1. Stakeholder engagement | Key stake holders in this work include national-level policy makers, particularly from areas with high OAE prevalence. A representative of the latter groups participated in the work and is co-author of the paper. | Author list, author contribution |
| 1. Complete model documentation | We used a previously published model, that has been described elsewhere in detail. We included a brief model description in the current paper, with references to original model description papers. A link to the publicly available source code is given in the section S1 of the Supplementary document. We provided full information on the quantification of all model parameters pertaining to transmission, life history and productivity of the parasite, morbidity, vector, drug efficacy, treatment histories (timing, frequency and coverage of mass treatment), and surveys. | Methods section; section S1 of the S1 Text |
| 1. Complete description of data used | This paper used empirical data to verify that model-predicted patterns in mf and OAE prevalence match to empirically observed patterns. A summary description of the study area and data is included in the methods section with references to earlier publications where relevant. Relevant summary data, to which model predictions are compared, are included in figures in the manuscript and supplement. | Methods section in the main text, Fig 2 main text |
| 1. Communicating uncertainty | We assessed the sensitivity of model outcomes to univariate or multivariate changes in key parameters of the OAE morbidity sub-model. | Figures S3-S5 in S1 Text |
| 1. Testable model outcomes | We tested whether the model-predicted age-specific OAE-mf prevalence association is in line with data from Maridi, and whether the model predicted association between overall mf prevalence and OAE prevalence is in line with data from a systematic review. For Maridi, we made predictions pertaining to the situation after the study period could be tested against data, should relevant data be collected. | Comparison of model predictions to data is shown in Figs 2 and 3. The results presented in figures 4-7 could be compared to data, should they become available |
| ^a^ Communication of adherence to the five principles of the NTD Modelling Consortium for policy-relevant work, described in [1].  ^b^ Full formulation of the principles:   1. Don't do it alone. Engage stakeholders throughout, from the formulation of questions to the discussions on the implications of the findings. 2. Reproducibility is key! Prepare and make available (preferably open-source) a complete technical documentation of all model code, mathematical formulas, assumptions and their justification, allowing others to reproduce the model. 3. Model calibration, goodness-of-fit and validation are fundamental processes of scientific modelling. All data used should be described in sufficient detail to allow the reader to assess the type and quality of these analyses. When using data by reference, use Principle 2. 4. Communicating uncertainty is a hallmark of good modelling practice. Perform a sensitivity analysis of all key parameters, and for each paper reporting model predictions include an uncertainty assessment of those model outputs within the paper. 5. Model outcomes should be articulated in the form of testable hypotheses. This allows comparison with other models and future events as part of the ongoing cycle of model improvement. | | |

References

[1] Behrend et al. 2020. Modelling for policy: The five principles of the Neglected Tropical Diseases Modelling Consortium. *PLoS Negl Trop Dis* 2020; **14**(4): e0008033.
