# Supplementary material for: Onchocerciasis-associated epilepsy in Maridi, South Sudan: Modelling and exploring the impact of control measures against river blindness
Source: PLoS Negl Trop Dis. 2023 May 26;17(5):e0011320. doi: 10.1371/journal.pntd.0011320 (PMC10249816; doi:10.1371/journal.pntd.0011320)
Supplement: Attachment — Submitted filename: Response to Reviewers_10APR.pdf [file pntd.0011320.s003.pdf]

RESPONSE TO REVIEWERS

Manuscript: ***Onchocerciasis-associated epilepsy in Maridi, South Sudan: Modelling and exploring the impact of control measures against river blindness (PNTD-D-23-00144)***

Reviewer #1

Bhattacharyya et al. applied a mathematical modelling (ONCHOSIM) to predict the impact of MDA and vector control on epidemiology of OAE in the Maridi region (South Sudan). Based on their results, the authors suggest that OAE incidence and prevalence can be reduced by onchocerciasis elimination programmes and conclude that these programmes need to be intensified to prevent OAE. The content of the manuscript and presented findings are very important and from broad interest for researchers and health officials/national NTD programmes. However, several questions remain uncertain and need to be addressed:

In regards to the model assumptions the authors stated: "We assume that brain damage is (directly or indirectly) induced by immune responses triggered by dying mf..." Ivermectin is a microfilaricidal drug inducing mf cell death. According to assumption of the authors this would mean that MDA induces brain damage and thus treated individuals have a higher Di (t)/OAE rate?

Response: We now omit the word "dying", as we believe that the mf can use various mechanisms to inflict brain damage (direct entry into the central nervous system, immune reactions, excretory secretory products, etc). Ivermectin does not induce brain damage, as it does cannot penetrate the central nervous system. Ivermectin MDA rather prevents OAE by reducing mf densities. A cohort study indeed showed that the risk to develop OAE is dependent on the intensity of onchocerciasis infection (*see Chesnais et al. The temporal relationship between onchocerciasis and epilepsy: a population-based cohort study. Lancet Inf Dis 2018;18: 1278–1286. doi:10.1016/S1473-3099(18)30425-0*)

2) Did the authors consider to include season (rain/dry season) into the model.

Response: The ONCHOSIM Model already accounted for seasonal variations in transmission patterns. The seasonal variation is reflected through variable fly biting pattern. South Sudan has a rainy season generally no more than eight to nine months a year, beginning as early as May and continuing as late as December. Other months of the year are usually dry and hot. The monthly fly biting rate varies according the weather. The mean monthly biting rate during rainy season is approximately 3000, which is 1.5 times higher compared to that of during dry season.

3) Since the authors conclude that MDA programmes need to be intensified, would ivermectin treatment twice a year improve OAE incidence?

Response: Increasing ivermectin frequency would certainly help as this will decrease the individual's microfilarial load to a greater extent but will also ensure higher coverage since some people who missed ivermectin distribution during one round can catch up during the second round. We have plotted the incidence comparing annual and biannual

implementation of MDA treatment. Please see Figure S6 in the supplementary information.

4) The authors need to check the references. Maybe, I missed it but refs 16 and 24 is not mentioned within the manuscript.
<u>Response</u>: Thank you for this remark. We have now corrected the numbering of the references.

Reviewer #2

This manuscript presents the results of a study that utilizes a well-established model for onchocerciasis to investigate the effect of the currently available interventions (ivermectin MDA and vector control) on the prevalence of onchocerciasis associated epilepsy (OAE). OAE has been a manifestation of *Onchocerca volvulus* infection that has received increasing attention over the past few years. The model used here (ONCHOSIM) is one of the two most well established models for onchocerciasis, and this study involved only minor modifications to the model to evaluate the effect of the interventions on OAE.
The results are clearly presented and are basically what someone knowledgeable in the field would have predicted a priori. Ivermectin MDA was predicted to reduce the prevalence of OAE prevalence fairly quickly, while vector control also reduced prevalence, although less rapidly. Combining both MDA and vector control led to the most rapid reduction of prevalence.
I had a few minor concerns that I would like to see the authors address:

The authors report that they used data from a single community in South Sudan to parameterize the model and then report that the model closely replicated the observed prevalence of OAE in that community. Using the same dataset to parameterize the model and then validating the model against the same data is circular. The authors only had the single dataset, and recognize this as a limitation in the conclusions. But I do not feel the validation was really sound and suggest deleting this.
<u>Response</u>: We agree with the reviewer and have now omitted the validation section. Data is currently being collected in South Sudan after implementing strengthening onchocerciasis elimination interventions, and we will use empirical data to validate this model in the future.

The authors report the effect of MDA at different coverages, but do not define coverage rates. In the field, two versions of coverage data are used - the percentage of the total population (the APOC standard) and the percentage of the eligible population treated (the Carter Center/OEPA standard). They need to define which of these they used.
<u>Response</u>: We defined coverage based on the entire study population, not just the eligible individuals. This has now been specified in the text: "Of note, the denominator used for the calculation of MDA coverage was the entire population (all ages included)."

The authors report the model predictions for OAE prevalence and mention that there is a shift in the prevalence age distribution as a result of OAE being a chronic condition and reducing the incidence through MDA and vector control would explain this shift. I would actually like to see the model predictions for incidence as well as prevalence.

<u>Response</u>: We have presented the sensitivity analysis of MDA implementation or vector control for incidence. Please see the supplementary information (Figure S4, S5. S6 and S7).

The conclusions are all well supported by the data and the limitations are discussed. Public health relevance is clearly discussed.
This manuscript represents the first attempt to examine the effect of the available interventions for onchocerciasis on OAE. Basically, the results suggest that the current interventions, if successfully implemented, will result in a dramatic reduction in the prevalence of OAE. This is reassuring to those of us involved in the effort to eliminate onchocerciasis in Africa.
<u>Response</u>: Thanks for the comment

Reviewer #3:

The study objectives are clearly articulated, -the study design is appropriate to address the stated objectives.

-study population is clearly described,

- the sample size is sufficient to ensure adequate power to address the hypothesis being tested.

-The statistical analysis used support the conclusions.

-There is no concerns about ethical or regulatory requirements

- results presented match the analysis plan

-The results are clearly presented,

- On page 15, Figure 6: the Y-axis should read New OAE prevalence (%) instead of OAE Prevalence (%) -Tables and figures are of sufficient quality and clarity
<u>Response</u>: Thank you for the remark, Figure 6 has been edited accordingly.

The conclusions are supported by the data presented

-the authors have presented the limitations of the analyses performed

- the helpfulness of the findings to advance the understanding of the topic under study is discussed -the public health relevance of the study is clearly highlighted

The development of a mathematical model to predict the impact of the control measures against onchocerciasis is very timing and highly relevant. During the recent years, evidence have accumulated in Sub Saharan Africa on the association between Onchocerciasis and Epilepsy. Studies have also demonstrated that the control of Onchocerciasis has direct impact in reducing the number of new cases of epilepsy in the endemic area of Onchocerciasis. The capacity of predicting the impact of control measures in different scenarios of treatment coverage or Simulium biting rate reduction was lacking to give to the health system a comprehensive tool to address the problem of epilepsy associated to Onchocerciasis. The findings from this study are filling the gap of knowledge.

Response: Thanks for the comment.
